# Functional Outcome of Transforaminal Lumbar Interbody Fusion Surgery in Spondylolisthesis: An Observational Study

**DOI:** 10.31729/jnma.9156

**Published:** 2025-07-31

**Authors:** Saroj Chandra Dahal, Sabin Pokharel, Surya Bajra Lama Waiba, Deepak Kaucha, Rajesh Kumar Chaudhary, Ram Krishna Barakoti, Babu Kaji Shrestha

**Affiliations:** 1 Department of Orthopedics, B&B Hospital, Gwarko, Lalitpur, Nepal; 2 Department of Orthopedics and Trauma Surgery, National Trauma Centre, National Academy of Medical Sciences, Mahaboudha, Kathmandu, Nepal

**Keywords:** *follow up studies*, *nepal*, *spinal fusion*, *spondylolisthesis*, *visual analogue pain scale*

## Abstract

**Introduction:**

Transforaminal lumbar interbody fusion is indicated for stabilizing and fusing spondylolisthesis. There is no uniformity in functional outcomes reported across different studies. We aimed to find the functional outcomes of transforaminal lumbar interbody fusion in our cases.

**Methods:**

This retrospective observational study included post-operative spondylolisthesis cases. Visual Analogue Scale and the validated Nepali Oswestry Disability Index (version 2.1a) were used to grade pain and functional outcomes. Data were entered in MS Excel 2016 and analysed using JASP version 0.19.3. Point estimates at 95% Confidence Interval were calculated, along with frequencies and percentages for binary data, and mean with standard deviation or median with interquartile range for continuous data.

**Results:**

Among the total 68 patients, the median post-operative duration of follow-up was 27 months (IQR 39), ranging from 6 to 80 months. Sixty-three (92.65%) cases belonged to lower grade (grade I and II) spondylolisthesis. Predominant radiculopathy accounted for 56 (82.35%) cases. A total 61 (89.71%) cases underwent single-level transforaminal lumbar interbody fusion, whereas remaining 7 (10.29%) had two-level transforaminal lumbar interbody fusion surgery. The VAS score mean was 2.09+1.05 (95% CI: 1.83 - 2.34), where 63 (92.65%) had mild pain and 5 (7.35%) cases had moderate symptoms at the final follow-up. The mean Oswestry Disability Index score was 9.57+3.81(95% CI: 8.65 - 10.49).

**Conclusions:**

Patients reported lower pain scores and better functional outcomes post-operative in transforaminal lumbar interbody fusion surgery.

## INTRODUCTION

Transforaminal lumbar interbody fusion (TLIF) is primarily indicated for the stabilization and arthrodesis of spinal deformity like spondylolisthesis and is known to restore the lumbar lordosis.^1^ The TLIF is a commonly performed surgery worldwide.^2,3^

In a study with one year follow up, the clinical outcomes improved after TLIF, pain on Visual Analogue Scale (VAS) decreased by 30.78 points and Oswestry Disability Index (ODI) decreased by 15.11 points, from preoperative levels.^4^ Similarly, another study reports decrease in ODI by 13.5 at one year follow up.^2^ Whereas, a recent prospective study done in Nepal showed statistically significant decrease in ODI by 42 in one year follow up post-operative TLIF surgery. ^5^

Since, there is disparity in findings, we aimed to find out the functional outcome in patients after the TLIF surgery in our cases. This would help to ascertain the long-term outcome of the procedure and make informed decision.

## METHODS

This was an observational study done on cases of spondylolisthesis who were operated at the study center and followed up at the Outpatient department (OPD) of department of Orthopedics. The study site was B&B Hospital, Gwarko, Nepal. The ethical clearance was taken from IRC of B&B Hospital with clearance number B&BIRC-24-59 Date 26^th^ January 2025. Convenience sampling technique was employed. The study period was for 3 months following the ethical clearance. All the cases more than 20 years of age having undergone transforaminal lumbar interbody fusion at the study center with the diagnosis of spondylolisthesis and consenting for the enrollment in the study were included in the study. Whereas, the cases having post traumatic or post infective spondylolisthesis, and cases with known diagnosis of spinal malignancy were excluded from the study as these would impact the functional outcome.

Sample size calculation was done using the following formula:

n=(Zα/2×σ)^2^/ Δ^2^ = 61, with 10% attrition rate the final sample size is 68.

Where, n = required sample size, Zα/2 = Z-score corresponding to 95% confidence level= 1.96, σ (standard deviation) = 19.9 from previous study by Mulvaney et al ^6^ for mean final follow up ODI score.

Δ = 5 % margin of error.

The cases presenting to the out-patient department (OPD) for their follow ups during the study period with at least three months postoperative duration after the surgery were interviewed by the principal investigator after gaining the consent for participation in the study. The demographic details, level of fusion, clinical symptoms at the time of surgery [predominant low back pain (LBP) and predominant radiculopathy (RAD)], diagnosis, duration of symptoms till time of surgery, type of spondylolisthesis according to Wiltse et al.^7^ and Meyerding grading^8^ were noted on proforma from the patient charts. The current pain was measured using the 11 point (0-10) Visual Analogue Scale (VAS) of grading for pain where 0= no pain, 1-3= mild pain, 4-7= moderate pain and 8-10= severe pain. The VAS scoring was measured on a 10 centimetres (cm) line with 1 cm (10 mm) to each point of the scale.^9,10^ The two endpoints correspond to ‘no pain’ and ‘worst possible pain’. Patient marked on the line representing the current level of back pain. The distance from ‘no pain’ end was measured, and scoring given out of 10. This VAS score was noted on proforma. The functional status was calculated using the cross culturally adapted and validated Nepali version of Oswestry Disability Index (ODI) which assessed the person ability to function in nine different activities of daily living accessed by 10 items. ^11-13^ The Nepali ODI version uses kilometres to ascertain the walking distance (section 4) and modified on ODI version 2.1a. The ODI is patient self-administered questionnaire. The ODI score was noted on the proforma.

The data were entered on MS Excel 2016 and analysis done on JASP version 0.19.3. The proportion and mean with standard deviation (SD) were used. For non-normally distributed data median with Interquartile range (IQR) was used. The point estimate at 95% Confidence interval (CI) was used.

## RESULTS

A total of 68 patients fulfilling the inclusion criteria presenting to the OPD for follow ups were enrolled in the study. The Median postoperative duration of follow up was 27 months (IQR 39), ranging from 6 to 80 months. The mean age at the time of surgery was 47.01 + 12.62 years ranging from 23 years to 73 years. The mean age of female and male was 47.82 + 12.32 and 44.59 +13.56, respectively. The cases had symptoms for a maximum of 120 months before the surgery where median time from start of symptoms to surgery was 19 months (IQR 24).

Males with predominant back pain as clinical feature presented in 11.60 + 2.30 months and female in 19.43+ 12.74 months in average. Similarly, male with radiculopathy presented in 26.83 + 20.46 months and female in 28.98 + 24.89 months in average. In this study, female participants were 51 (75%). Out of total participants 56 (82.35%) presented with predominant radiculopathy before surgery, 44 (64.71%) had degenerative spondylolisthesis type and 40 (58.83%) had Meyerding’s Grade II slip (Table 1).

**Table 1:** Demographic features of the patients with spondylolisthesis following transforaminal lumbar interbody fusion surgery (n= 68).

Variables	n(%)
Sex	
Female	51(75)
Male	17(25)
Clinical presentation before surgery	
Predominant back pain	12(17.65)
Predominant radiculopathy	56(82.35)
Spondylolisthesis types	
Degenerative	44(64.71)
Isthmic	22(32.35)
Dysplastic	1(1.47)
Post-surgical	1(1.47)
Meyerding’s Grading	
Grade I (0-25% slip)	23(33.82)
Grade II (25-50% slip)	40(58.83)
Grade III (50-75% slip)	4(5.88)
Grade IV (75-100% slip)	1(1.47)
Grade V (>100% slip)	0(0)

There were 34 (50%) female and 10 (15.63%) male having degenerative causes. Similarly, there were 16 (23.53 %) female and 6 (8.82%) male having isthmic type spondylolisthesis. There were 61 (89.71%) cases who underwent single level fusion and 7 (10.29%) cases had two levels TLIF surgery.

**Figure 1:**
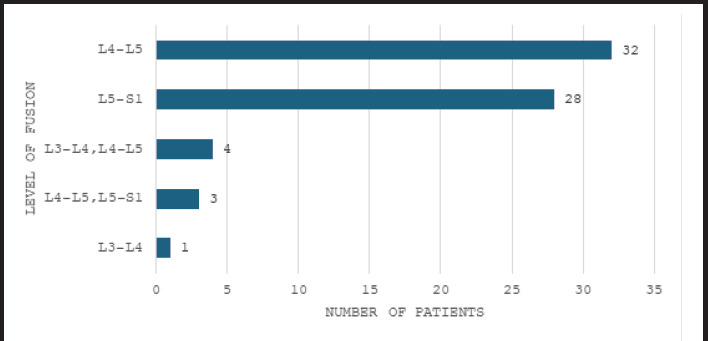
Levels of fusion in patients with spondylolisthesis following transforaminal lumbar interbody fusion surgery (n= 68).

**Figure 2:**
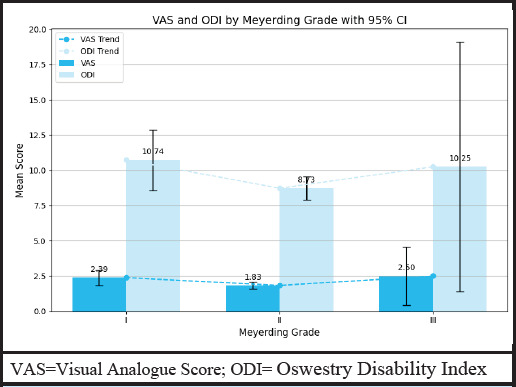
VAS and ODI by Meyerding grade with 95% CI in spondylolisthesis following transforaminal lumbar interbody fusion surgery (n= 68).

Thirty-two (47.06%) cases had undergone TLIF at L4-L5 level (Figure 1). The mean VAS score was 2.09+1.05 and the mean ODI score was 9.57+3.81 (Table 2). No case of severe or crippling back pain was present post-operative at final follow up while 65 (95.59%) were graded as minimal in disability index (Table 3). There was one case in Meyerding’s grade IV, the mean VAS score for Meyerding grade II was 1.83+0.78 and that for grade III was 8.73+2.55 (Figure 2).

**Table 2:** Visual analogue and Oswestry disability index scores in patients with spondylolisthesis following transforaminal lumbar interbody fusion surgery (n= 68).

Variables	Minimum score	Maximum score	Mean+SD	95% CI
VAS	1	5	2.09+1.05	1.83-2.34
ODI	5	24	9.57+3.81	8.65-10.49

**Table 3:** Visual analogue score and Oswestry disability index gradings in patients with spondylolisthesis following transforaminal lumbar interbody fusion surgery (n= 68).

Variables	n(%)
Visual Analogue Score	
No pain (0)	0(0)
Mild pain (1-3)	63(92.65)
Moderate pain (4-6)	5(7.35)
Severe pain (7-10)	0(0)
Oswestry Disability Index	
Minimum (0-20)	65(95.59)
Moderate (21-40)	3(4.41)
Severe (41-60)	0(0)
Crippling (61-80)	0(0)
Bed-bound (80-100)	0(0)

## DISCUSSION

Transforaminal lumbar interbody fusion (TLIF ) is a commonly performed procedure for spondylolisthesis and has shown better VAS, ODI score, clinical satisfaction of the patient and fusion rates than other forms of fusion surgeries. ^1,14,15^ In our study the mean VAS and ODI score after the TLIF surgery for spondylolisthesis were 2.09 (SD 1.05) and 9.57 (SD 3.81) respectively. Among them 63 (92.65) had mild pain and 65 (95.59) had minimal disability. This shows the effectiveness of attaining low pain level and higher functional status of patients after the TLIF surgery.

In our study 51 (75%) of the cases of the spondylolisthesis were female. A systematic review with focus on gender specific prevalence of lumbar degenerative spondylolisthesis found that the female to male ratio is higher in cases undergoing surgery (2:1) than the general population (1.3 : 1).^16^ Our cases were also the one who had undergone surgery and F:M is 3:1. This may be due to males trying to avoid surgery due to financial reasons in a private institutions and opting for conservative management. It has also been found that females more often received surgical treatment then males which is the similar findings in our study. ^16^ In the cohort from Iran nearly 60% of the patients were females which is comparable to our findings.^17^

Previous studies have shown improvement of patient pain scores after the TLIF surgeries. A meta-analysis of 16 studies with 697 patients with TLIF surgery included showed decreased VAS score post operatively. ^14^ A study on 27 patients post-operative minimally invasive TLIF showed VAS of 1.1 ± 0.8 on 6 months follow up. ^18^ Djurasovic et al showed that 100 points VAS decreased by 27.03 from preoperative level on final follow up. ^4^ In the same study the VAS decreased to 41.25 on 1 year follow up but again increased slightly to 45 on 2 year follow up, so the pain may increase later on. ^4^ Our study had median follow up time of over 2 years but the pain VAS was lower 2.09 on 11point scale VAS. The difference may be due to recall bias of retrospective study, we took the VAS scoring at the follow up visit so there is no recall bias. Another meta-analysis included 273 patients and found mean 2.97 VAS score at final follow up. ^19^ Another study in Nepalese comparable population showed 2.63 VAS for back pain on 1 year follow up.^20^ With more than 2 years follow up, Dahal et al showed 1.59 mean VAS score in a retrospective study on Nepalese population. ^21^ Another study showed 3.7 mean VAS score on 60 months follow up.^22^ So the TLIF has good post-operative pain scores and relieves the pain due to spondylolisthesis.

Similarly, for the functional scoring with ODI, the literatures suggest decrease in it post operatively. Gurung et al showed gradual decrease in ODI score on 2,6, and 12 months follow up with final 17.36 score. ^5^ The mean ODI score decreased from 46.7 to 20.7 on one year post-operative follow up in a study.^23^ Poudel et al had improvement to 23.96 ODI on mean follow up of 13.56±7.15 months.^20^ Sixty months follow up had 24.8±19.0 ODI score in a study.^22^ At 6 months the score improved to 15.7±1.3 in another study. ^18^ In Nepalese population studied by Bijukachhe and team, the post-operative ODI was 20 on the final follow up of 27. 47 + 17.62 months. ^21^ A meta-analysis had four articles reporting on the ODI post-operative TLIF and found it to be 27.85±24.81.^19^ At 2 years follow up the score decreased to 29.74 in another study. ^4^ One level TLIF on 183 patients at mean 3.57 years follow had ODI of 28.4 (SD 19.8).^2^ We had the mean ODI score of 9.57 (SD 3.81) in our study. All the above-mentioned studies show that ODI decreases post operatively TLIF but our findings were better. But we did not have the baseline values to ascertain the findings. But we had range of ODI from 5 to 24. We used the population specific validated ODI scoring which could have increased the understanding of questions causing the better result. Overall, TLIF has lower disability scoring at the follow up and functional outcome is good.

We used the convenience sampling method and could not randomize the cases so the generalizability of data may be questionable. We used the SD for calculation of the sample size from another study but our SD was lower, this could be because of lower variability in our data which has increased precision of our effect estimates. The preoperative scores were not noted so the amount of decrease in the scoring could not be documented. But we used the postoperative scoring at the follow up visit hence decreasing the recall bias. To minimize the selection bias, we included the cases with at least 3 months post-operative follow up. Also, we had longer follow-up which could help to find the long-term outcome. We used the validated Nepali version ODI scoring so that patients understood the questions and give appropriate answers. Future studies with comparison of preoperative and post-operative scoring with the data on change on lumbar lordosis, fusion rates and complications is necessary. Also, comparing the outcomes with the other fusion techniques will help in better understanding the result of surgery and choose the better one for the patient.

## CONCLUSIONS

Patients reported lower pain scores and better functional outcomes post-operative transforaminal lumbar interbody fusion. The back pain was mild, and disability was minimal at the final follow up relieving the patient of discomfort.

## Data Availability

The data are available from the corresponding author upon reasonable request.
